# Metabolic acidosis of chronic kidney disease and subclinical cardiovascular disease markers: Friend or foe?

**DOI:** 10.1097/MD.0000000000008802

**Published:** 2017-11-27

**Authors:** Cristina Căpuşă, Gabriel Ştefan, Simona Stancu, Mariana Lipan, Lilach Daniel Tsur, Gabriel Mircescu

**Affiliations:** a Nephrology Department, “Carol Davila” University of Medicine and Pharmacy; b “Dr Carol Davila” Teaching Hospital of Nephrology; c “Carol Davila” University of Medicine and Pharmacy; d Romanian Renal Registry, Bucharest, Romania.

**Keywords:** cardiovascular disease, markers of subclinical cardiovascular disease, metabolic acidosis of chronic kidney disease, vascular calcifications

## Abstract

The effect of chronic metabolic acidosis (MA) on cardiovascular disease (CVD) in the setting of chronic kidney disease (CKD) is largely unknown. Therefore, we aimed to study this relationship in nondialysis CKD patients.

This cross-sectional, single-center study prospectively enrolled 95 clinically stable CKD patients (median age 61 (58, 65) years, 60% male, median eGFR 27 (22, 32) mL/min). Data on CKD etiology, CVD history, CVD traditional, and nontraditional risk factors were obtained. Also, markers of subclinical CVD were assessed: intima-media thickness (IMT), abdominal aortic calcifications (Kauppila score—AACs), cardio-ankle vascular index (CAVI), ankle-brachial index (ABI), ejection fraction, and interventricular septum thickness. Using the serum bicarbonate cutoff value of 22 mEq/L, comparisons between MA (<22 mEq/L; 43 patients) and non-MA (≥22 mEq/L; 52 patients) groups were performed.

Vascular (40%), tubulointerstitial (24%), and glomerular (22%) nephropathies were the main causes of CKD. Twenty-three percent of patients had diabetes mellitus, but only 5% were considered to have diabetic nephropathy. Patients with chronic MA had lower eGFR (*P* < .01), higher iPTH (*P* = .01), higher serum phosphate (*P* < .01), and increased serum cholesterol (*P* = .04) and triglycerides (*P* = .01).

Higher ABI (*P* = .04), lower IMT (*P* = .03), CAVI (*P* = .05), and AACs (*P* = .03) were found in patients with chronic MA.

Separate binomial logistic regression models were performed using ABI (cutoff 0.9), CAVI (cutoff 9), IMT (cutoff 0.1 cm), and AACs (cutoff 1) as dependent variables. MA was used as independent variable and adjustments were made for iPTH, serum phosphate, eGFR, proteinuria, cholesterol, triglycerides, CVD score. The absence of MA was retained as an independent predictor only for the presence of AACs.

In conclusion, the present study shows a potential advantageous effect of MA on vascular calcifications in predialysis CKD patients. Thus, a guideline relaxation of the serum bicarbonate target might prove to be beneficial in CKD patients at high risk of vascular calcifications. However, one should always consider the negative effects of MA. Therefore, additional research is warranted before any clear clinical recommendation.

## 1 Introduction

Cardiovascular disease (CVD) is the leading cause of morbidity and mortality in chronic kidney disease (CKD) patients.^[1]^ Moreover, CVD often begins before end-stage renal disease (ESRD), and patients with reduced renal function are more likely to die of CVD than to develop ESRD.^[2]^ There are 3 pathological forms of CVD that should be considered in patients with CKD: alterations in cardiac geometry, including left ventricular hypertrophy, atherosclerosis, and arteriosclerosis.^[2]^ The clinical interventions on traditional and nontraditional risk factors for CVD were not as effective as in the general population, mainly because of the unique CKD setting.^[3,4]^ Therefore, identification of new risk factors may prove useful in the development of new targeted therapies.

Chronic metabolic acidosis (MA) is a common complication of CKD and, furthermore, lower serum bicarbonate levels have been associated with CKD progression. Moreover, the Kidney Disease Improving Global Outcomes (KDIGO) Clinical Practice Guideline for the Evaluation and Management of CKD suggest that patients with serum bicarbonate concentrations under 22 mEq/L should be treated with oral bicarbonate supplementation in order to maintain serum bicarbonate within the normal range. Although, there is an increased risk of death with metabolic acidosis of CKD (MAC), the effect of MAC on CVD is a subject of debate.^[5]^ Thus, in some studies MAC was associated with factors that could lead to CVD like hypertension and chronic inflammation, while in another study serum bicarbonate was not related to cardiac structural and functional abnormalities.^[6–8]^

Therefore, we aimed to investigate the relationship between MAC and markers of cardiovascular damage that address the 3 CVD pathological forms in nondialysis CKD patients.

## 2 Materials and methods

### 2.1 Subjects

This cross-sectional, single-center study prospectively enrolled 95 clinically stable CKD patients selected from those admitted to the Dr. Carol Davila Teaching Hospital of Nephrology in a 6-month period. Patients were invited to participate in the study if they were over 18 years of age, had an estimated glomerular filtration rate (eGFR) < 60 mL/min/1.73 m^2^ (abbreviated modification of diet in renal disease (MDRD) equation) on 2 occasions in a 3-month period. Patients with ESRD, those who received sodium bicarbonate and those who received any form of treatment for secondary hyperparathyroidism were excluded.

The study protocol was approved by the local Ethics Committee. All subjects signed an informed consent form before any study procedure.

### 2.2 Methods

Systematic data were obtained for every patient using a standardized questionnaire from the medical records, which included: CVD history (coronary artery disease-0/1, peripheral artery disease-0/1, cerebrovascular disease-0/1—we used a CVD score which was the sum of all cardiovascular comorbidities); traditional cardiovascular risk factors such as age, gender, body mass index (BMI), smoking status, hypertension (defined as a blood pressure ≥140/90 mm Hg in nondiabetics, ≥130/80 mm Hg in diabetics or use of antihypertensive medication), diabetes mellitus (defined as a plasma fasting glucose >126 mg/dL, a nonfasting glucose >200 mg/dL, use of insulin or antidiabetic medication), lipid profile (cholesterol, triglycerides); nontraditional cardiovascular risk factors—CKD (etiology, eGFR, proteinuria, albuminuria), calcium–phosphate metabolism parameters (serum parathyroid hormone (iPTH), vitamin D (25(OH)D), total and ionized calcium, phosphate, total alkaline phosphatase), inflammation (C-reactive protein (CRP), serum albumin), and serum uric acid.

Serum bicarbonate was measured using a clinical blood gas analyzer (Rapidpoint 500, Siemens Healthineers, Norwood, MA) at the admission time. The standard bicarbonate value (bicarbonate concentration in the plasma from blood which is equilibrated with a normal PaCO_2_ (40 mm Hg) and a normal PaO_2_ (over 100 mm Hg) at 37°C temperature) was used for analysis. The present study examined serum bicarbonate continuously per 1 mEq/L, and categorically using the cutoff value of 22 mEq/L based on the current recommended KDIGO Clinical Practice Guideline for the Evaluation and Management of CKD and the available literature.^[8,9]^

The following markers of subclinical CVD were assessed:

Intima-media thickness (IMT) determination was done using B-mode ultrasonography imaging of the carotid with a transducer frequency of 7 MHz. Up to 4 cm of the common carotid artery, the carotid bifurcation and the internal carotid 2 cm distally from bifurcation were scanned bilaterally using longitudinal and transverse sections. IMT was defined as the distance between the leading edge of the first echogenic line (lumen–intima interface) and the second echogenic line (media–adventitia interface) in plaque-free arterial segments. All measurements were performed under blind conditions.^[10]^

Abdominal aortic calcifications (Kauppila score—AACs) were evaluated on a lateral lumbar X-ray (acquired in the standing position) using a semiquantitative score described by Kauppila et al.^[11]^ First, the abdominal aorta in front of the first 4 lumbar vertebrae was identified. Then, points were assigned from 0 to 3 according to the length of each calcified plaque (0—absence of calcification, 1—calcification length on less than a third of the vertebral body, 2—calcification length between 1/3 and 2/3 of the vertebral body, 3—calcification length more than 2/3 of the vertebral body) identified along the anterior and posterior profile of the aorta in front of each of the lumbar vertebra taken into consideration. Thus, the score could vary from a minimum of 0 to a maximum of 24 points.^[11]^ The measurements were performed by an independent examiner who was unaware of the patient's characteristics or the purpose of the study.

The ankle-brachial index (ABI)—an index of atherosclerotic peripheral vascular disease—and cardio-ankle vascular index (CAVI)—a marker of arterial stiffness—were measured with the subjects in supine position at rest for at least 10 minutes by a trained technician using the VaSera VS-1000 screening device (Fukuda Denshi, Tokyo, Japan) as described in the manufacturer's protocol.

Ejection fraction (EF) was calculated using the Simpson method in 2D mode echocardiography as (end-diastolic volume—end-systolic volume)/end-diastolic volume. Left ventricular systolic dysfunction was defined as EF <30%.^[12]^

Interventricular septum thickness was assessed in diastole using 2-dimensional M-mode echocardiography with a 2.5 MHz transducer.

### 2.3 Statistical analysis

Continuous variables are presented as mean or median and 95% confidence interval, according to their distribution, and categorical variables as percentages. The mean of bilateral CAVI, ABI, and IMT values were used in analysis. Using the serum bicarbonate cutoff value of 22 mEq/L, comparisons between MAC (<22 mEq/L) and non-MAC (≥22 mEq/L) groups were performed with Student *t* test, *χ*^2^ test, Mann–Whitney *U* test, and the Kruskal–Wallis test, as appropriate. The Spearman test was used to assess correlations.

Multivariable-adjusted binomial logistic regression analyses were performed in separate models using ABI (higher or lower than 0.9), CAVI (higher or lower than 9), IMT (higher or lower than 0.1 cm), AAC (higher or lower than 1), EF (higher or lower than 30%), and IVS thickness (higher or lower than 10 mm) as the dependent variable.^[13–15]^ Serum bicarbonate concentration was used as an independent variable and adjustments were made for variables that had a significant univariate test, that is, iPTH, serum phosphate, eGFR, proteinuria, cholesterol, triglycerides. CVD score was also included in the models as independent variable in order to control any possible confounding effect. Backward stepwise Wald model selection was used in the multivariate analysis. For the regression analysis, variables were log transformed in order to satisfy assumptions of normality.

A *P* < .05 was considered statistically significant. Analyse-it (Analyse-it Software, Ltd, Leeds, UK) and SPSS (SPSS, Inc., Chicago, IL) software were used to analyze the data.

## 3 Results

The studied population had a median age of 61 (58, 65) years, and 60% were male. Most of the patients were in stage 3B or 4 CKD (32% and 34%). Vascular (40%), tubulointerstitial (24%), and glomerular (22%) nephropathies were the main causes of CKD. Twenty-three percent of patients had diabetes mellitus, but only 5% were considered to have diabetic nephropathy. Arterial hypertension was found in 83% of the studied subjects. Ninety percent of the patients were treated for arterial hypertension: 75% received a renin-angiotensin system blocker (angiotensin-converting enzyme inhibitors or angiotensin receptor blockers), 56% received β-blockers, 30% calcium channel blockers, and 20% diuretic therapy.

**Figure 1:**
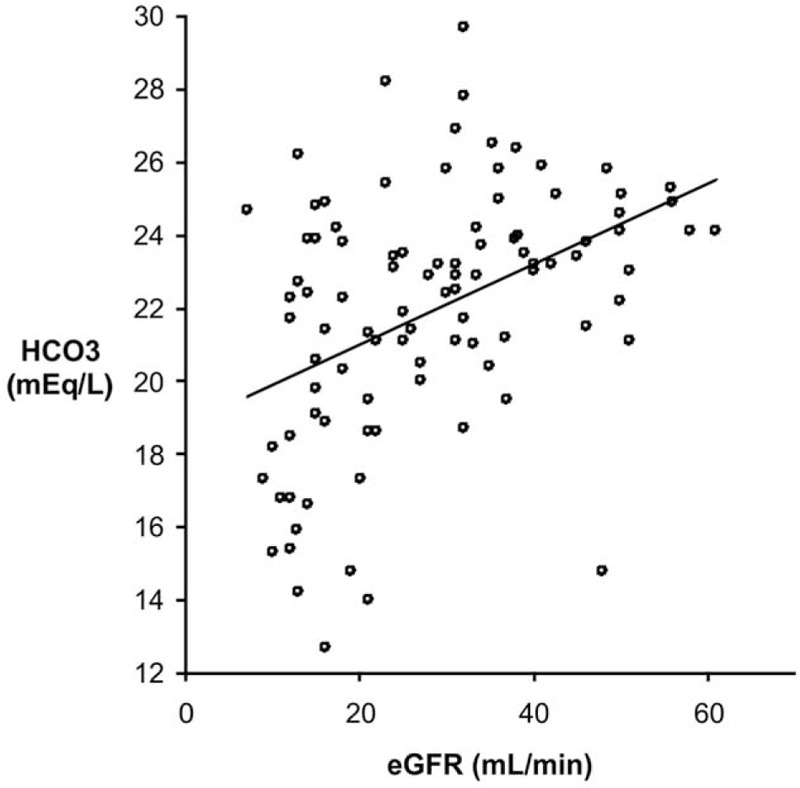
Correlation between serum bicarbonate and eGFR.

Serum bicarbonate had a median value of 22.7 (95% CI: 21.5, 23.2) mEq/L; and its levels decreased with the eGFR (*r*s = 0.46; *P* < .001) (Fig. 1). Forty-three percent of the studied patients had MAC; they were in more advanced CKD stages as suggested by the lower eGFR and higher proteinuria. Regarding the traditional risk factors of atherosclerosis, only serum cholesterol and triglycerides were higher in patients with MAC. Also, increased iPTH and serum phosphate levels were found in these patients (Table 1).

**Table 1:**
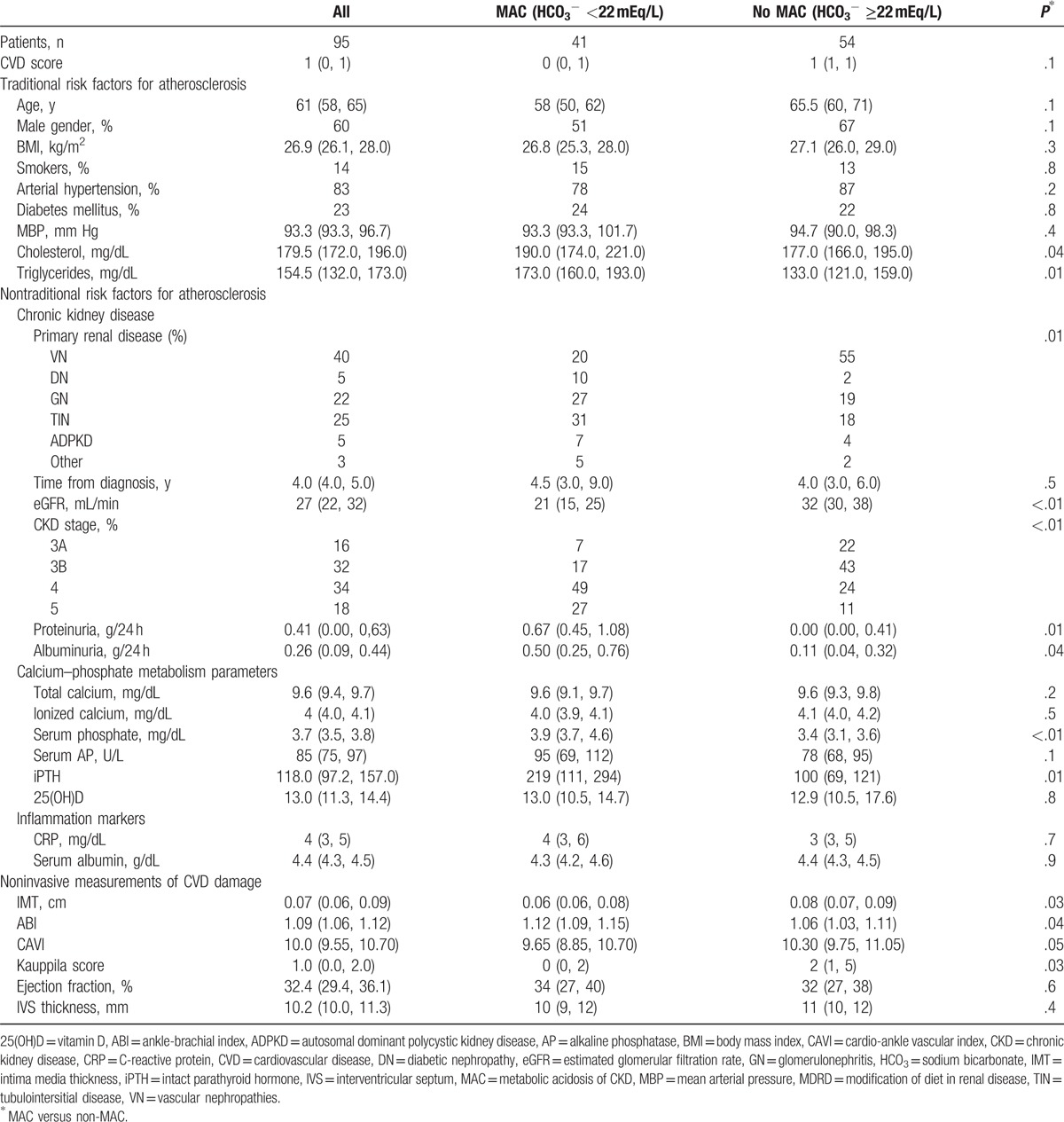
Investigated parameters in study groups.

**Figure 2:**
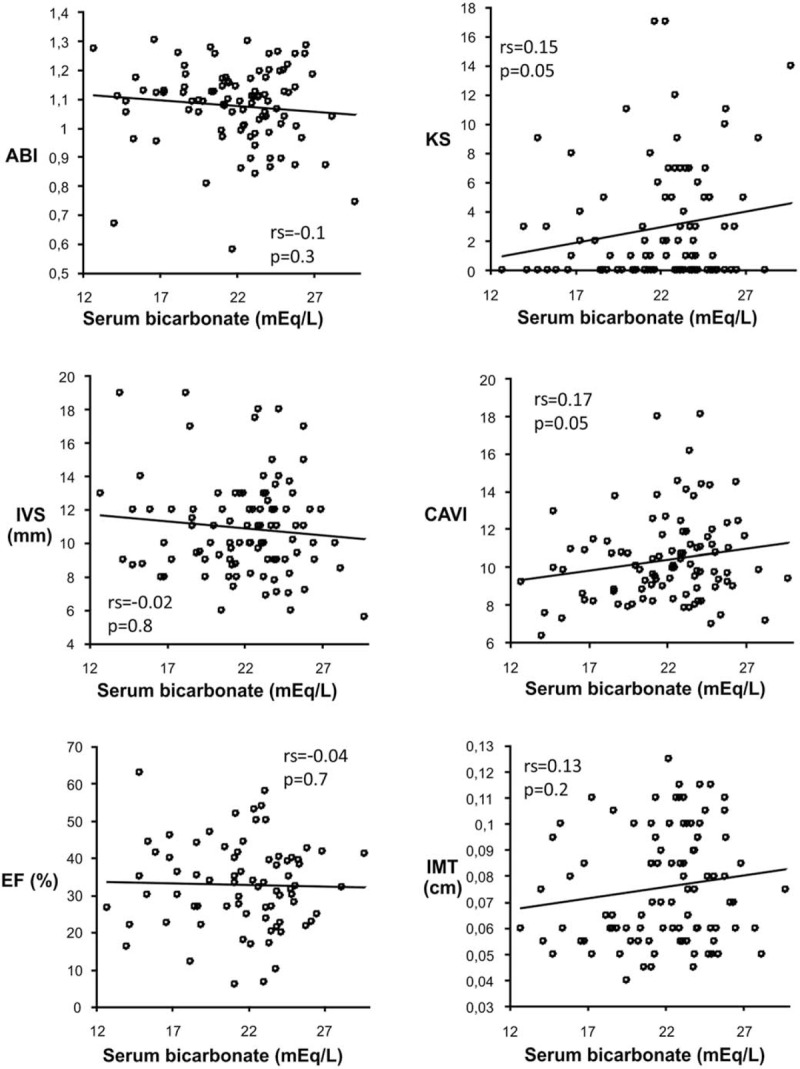
Correlations between noninvasive measurements of CVD damage and serum bicarbonate. ABI = ankle brachial index, CAVI = cardio-ankle vascular index, EF = ejection fraction, IMT, intima media thickness, IVS = interventricular septum, KS = Kauppila score.

In the univariate analysis of noninvasive markers of CVD, a potential beneficial effect of MAC on arterial disease was observed since these patients had lower IMT, CAVI, KS, and higher ABI (Table 1). However, only CAVI and KS increased with the serum bicarbonate level (Fig. 2).

The association between CVD subclinical markers and MAC was further evaluated in separate multivariable binomial logistic regression models adjusted for CVD risk factors. The absence of MAC was retained as an independent predictor only for the presence of AACs; the final model predicted 26% of the variability of the AACs (Table 2 ).

**Table 2:**
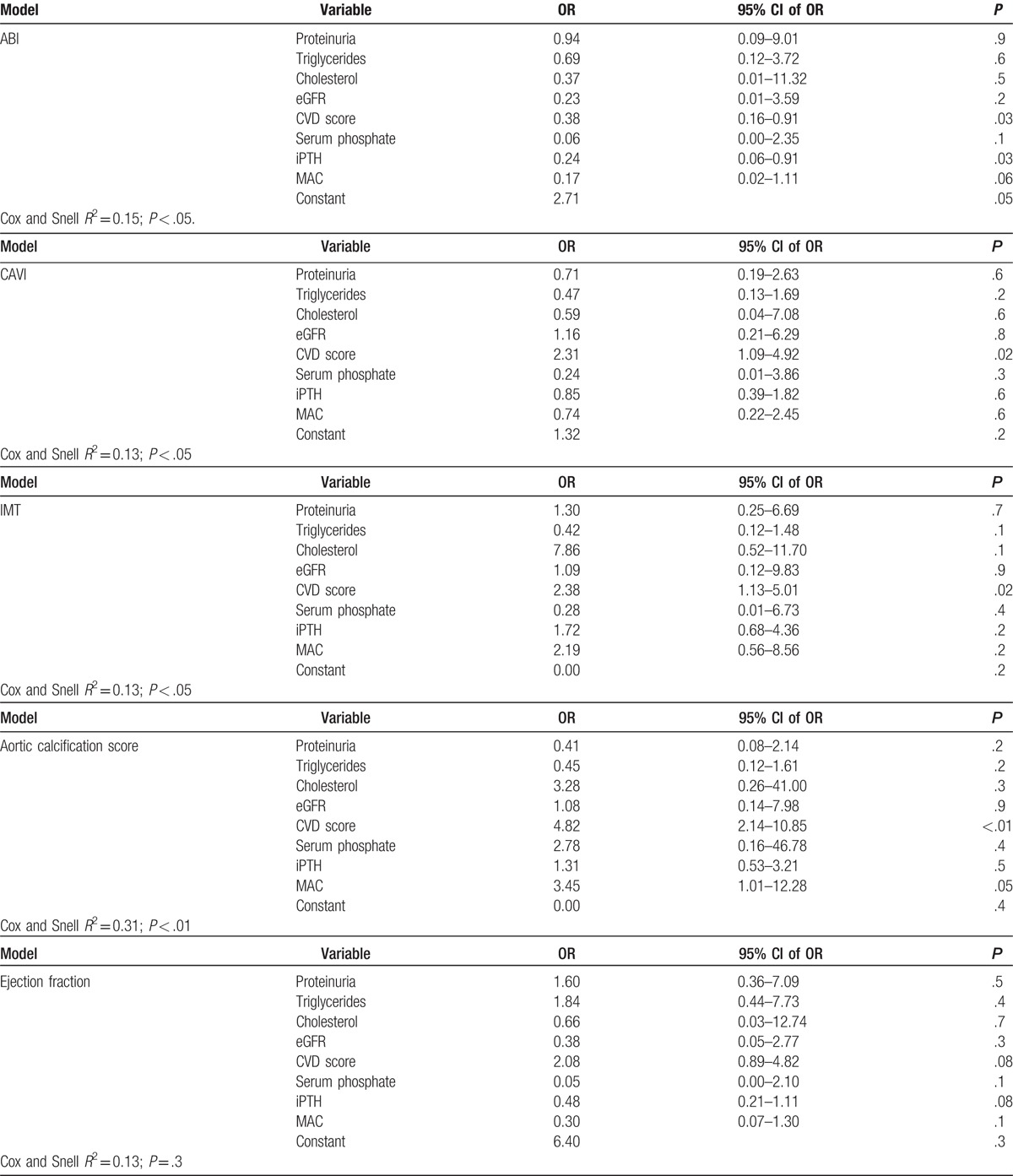
Relationship between the 6 studied noninvasive markers of cardiovascular damage and the cardiovascular risk factors in separate models of multivariable-adjusted binary logistic regression analysis (each model included the same variables; variables entered at step 1 for each model: MAC, iPTH, serum phosphate, eGFR, proteinuria, cholesterol, triglycerides, CVD score).

**Table 2 (Continued):**
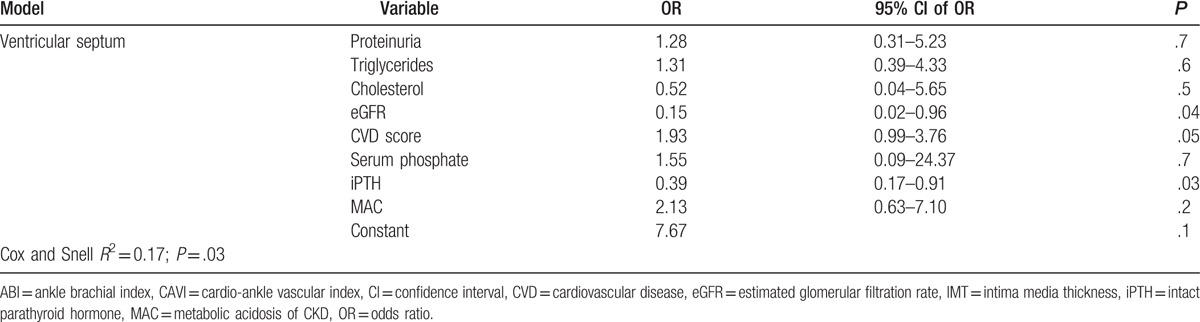
Relationship between the 6 studied noninvasive markers of cardiovascular damage and the cardiovascular risk factors in separate models of multivariable-adjusted binary logistic regression analysis (each model included the same variables; variables entered at step 1 for each model: MAC, iPTH, serum phosphate, eGFR, proteinuria, cholesterol, triglycerides, CVD score).

## 4 Discussion

In the present observational prospective study, we found that MAC might have a beneficial effect on vascular calcifications in nondialysis CKD patients. Moreover, this relationship remained after traditional and nontraditional cardiovascular risk factors adjustments. To the best of our knowledge, this is the first study to evaluate the association between serum bicarbonate and a large number of cardiovascular markers that address the full spectrum of CVD in nondialysis CKD patients.

Patients with CKD have a markedly increased incidence of cardiovascular events and mortality compared with the general population.^[16]^ Traditional and nontraditional risk factors contribute to the development of CVD in CKD patients. In earlier stages of CKD the contribution of traditional risk factors appears to be more important.^[17]^ Unique risk factors associated with moderate and severe CKD include uremic toxins, anemia, elevated levels of certain cytokines, abnormalities in bone mineral metabolism, malnutrition–inflammation complex syndrome.^[17,18]^ However, despite being a common complication of CKD, the impact of MA on cardiovascular function has been less studied.

A potential link between MAC and CVD could be the activation of inflammatory and neurohumoral mechanisms. Epidemiological studies showed that acidosis and inflammation are strongly linked in CKD patients.^[19,20]^ In experimental models, exposure of epithelial and macrophages to reductions in pH lead to increased inflammatory markers and development of atherosclerotic CVD.^[7,21]^ Furthermore, MA leads to enhanced production of catecholamines, endothelin-1, and aldosterone; which can contribute to changes in left ventricular mass and geometry.^[22]^ However, in our study we found no relationship between MAC and inflammatory markers. Moreover, serum bicarbonate level was not associated with ventricular septum thickness and EF. These results are in line with Dobre et al,^[8]^ who in a cohort of 3483 CKD patients found no independent association between serum bicarbonate and cardiac structural and functional abnormalities (i.e., left ventricular hypertrophy, left ventricular mass indexed to height, left ventricular geometry, EF, and diastolic dysfunction).

A number of studies reported that MA is related to higher prevalence of hypertension. Thus, in nonobsese adult women, higher serum bicarbonate was associated with lower prevalence of hypertension after adjusting for matching factors.^[23]^ Furthermore, hypertension was positively correlated with increased dietary acid load in adults and children.^[23,24]^ These data suggest that increased tissue acidity could be a risk factor for development of hypertension. However, in the Rotterdam study, Engberink et al^[25]^ found no evidence of an association between dietary acid load and risk of hypertension in older adults (≥55 years old). In our data, most of the patients had the diagnosis of hypertension, thus the lack of difference between the studied groups could be subjected to bias. Also, blood pressure comparison might have been biased by the therapy, since the majority of our patients were treated.

Concerning the investigated traditional risk factors for atherosclerosis, patients with MAC had higher levels of serum cholesterol and triglycerides. Interestingly, in a small uncontrolled study of patients undergoing hemodialysis, correction of MA was associated with significant decrease in serum triglycerides level, suggesting that MA may play a role in hypertriglyceridemia.^[26,27]^

ESRD patients who are on dialysis have a high prevalence of arterial calcifications which are associated with cardiovascular mortality.^[28]^ Also, there is a high prevalence of vascular calcifications in predialysis patients, which suggests that this process starts early in the course of the disease.^[29,30]^ Medial calcifications, also called arteriosclerosis, are the most frequent form of vascular calcifications in CKD patients.^[31]^ The process of arteriosclerosis consists in transdifferentiation of vascular smooth muscle cells of the media to osteoblastic cells, which further lead to osteoid tissue formation in the arterial wall.^[32]^ Bone exposure to MAC promotes bone dissolution: directly by increasing calcium release from hydroxyapatite and indirectly by osteoclast activity stimulation.^[33,34]^ Therefore, the question of the protective effect of acidosis on extraskeletal calcifications was raised.

In vitro data, from a rat aorta model of arteriosclerosis demonstrate decreased medial mineral deposition at low pH.^[35]^ In vivo, Mendoza et al^[36]^ investigated the influence of MA on the development of calcitriol-induced extraosseous calcifications in a rat model of uremia. They showed that acidosis caused by ingestion of NH_4_Cl prevents the development of extraskeletal calcifications in uremic rats treated with calcitriol, even though the plasma Ca and P were increased in these rats.^[36]^ Phosphate uptake through the sodium-dependent phosphate cotransporter (Pit-1) has been reported to be essential for vascular smooth muscle cells transdifferentiation in osteoblastic cells. In the same study, Mendoza et al^[36]^ showed that vascular calcification was associated with increased Pit-1 expression in aortic tissue and that acidosis prevented the upregulation of Pit-1. Thus, acidosis may inhibit the transdifferentiation of vascular smooth muscle cells by preventing the upregulation of Pit-1. In line with these experimental models we found a lower abdominal calcification score in patients with MAC. Furthermore, CAVI a marker of arterial stiffness increased with serum bicarbonate level. Since the process of vascular calcification seems to recapitulate osteogenesis and MAC impairs bone mineralization, these results suggests that MAC might attenuate the vascular calcification process in nondialysis CKD patients.

Other experimental studies suggest that MA can inhibit the activity of the already transdifferentiated osteoblast. Thus, MA has been reported to inhibit the production of collagen, to downregulate alkaline phosphatase and upregulate matrix-gla protein (an inhibitory protein of vascular calcifications) in cultured rat osteoblasts.^[37,38]^ Therefore, a potential advantageous effect of MA is maintained in all stages of vascular calcifications.

Interestingly, clinical studies on calciphylaxis indicate that sodium thiosulfate, an acidifying agent and a potent antioxidant, may be useful in the treatment of extraosseous calcifications in uremic and nonuremic patients.^[39–41]^

High PTH levels implicate rapid bone turnover with associated calcium and phosphorus release into the circulation which are further linked to vascular calcifications.^[42]^ Our patients with MAC had higher level of PTH, which could be due to the direct stimulatory effect of acidosis on PTH secretion.^[43]^ However, our data indicate that the potential beneficial effect of acidosis on vascular calcifications is present even in the face of high PTH and phosphate levels.

Vascular calcifications of the main arteries are associated with an ABI <0.9, while calcification of peripheral and distal arteries are associated with values >1.3.^[44]^ No ABI >1.3 was found in our study and median values were around 1, suggesting dominant calcification of the main arteries. Abnormal ABI and increased carotid IMT are independent predictors of cardiovascular morbidity and mortality in CKD patients.^[45,46]^ In univariate analysis, patients with MAC had lower ABI and IMT as compared with their counterparts. However, in the multivariable binomial logistic regression models adjusted for CVD risk factors MAC was not retained as an independent predictor for ABI and IMT.

MA has been linked to increases in tissue aldosterone, endothelin, and angiotensin II levels, mediators which have been proved to lead to CKD progression.^[47,48]^ Furthermore, increased ammonium production due to MAC is associated with local complement activation and increased tubulointerstitial fibrosis.^[49]^ Several observational studies have indicated that lower serum bicarbonate levels are associated with CKD progression and increased incidence of ESRD.^[9,50–52]^ Accordingly, in our population patients with MA were in more advanced CKD stages.

Some limitations of this study should be acknowledged. First, the data resulted from a cross-sectional design with a small sample size from a single center, which can limit the statistical power. However, the final cohort reasonably describes the CKD patients seen in day-to-day practice. Second, our results can only suggest a protective effect of MAC on arterial disease and not prove causality. Third, due to the design of the study the time line relation between CVD and CKD diagnosis was not studied. Fourth, the serum bicarbonate was calculated from pH and PaCO_2_ values using the Henderson–Hasselbach equation and not enzymatically measured.

In conclusion, the present study shows a potential advantageous effect of MA on vascular calcifications in predialysis CKD patients. Thus, a guideline relaxation of the serum bicarbonate target might prove to be beneficial in CKD patients at high risk of vascular calcifications. However, one should always consider the negative effects of MA. Therefore, additional randomized controlled trials are warranted before any clear clinical recommendation.
